# Modelling the effect of seasonal influenza vaccination on the risk of pandemic influenza infection

**DOI:** 10.1186/1471-2458-11-S1-S11

**Published:** 2011-02-25

**Authors:** Geoffry N Mercer, Steven I Barry, Heath Kelly

**Affiliations:** 1 National Centre for Epidemiology and Population Health, Australian National University, Canberra, Australia; 2 Victorian Infectious Diseases Reference Laboratory, Melbourne, Australia

## Abstract

**Background:**

Recent studies have suggested that vaccination with seasonal influenza vaccine resulted in an apparent higher risk of infection with pandemic influenza H1N1 2009. A simple mathematical model incorporating strain competition and a hypothesised temporary strain-transcending immunity is constructed to investigate this observation. The model assumes that seasonal vaccine has no effect on the risk of infection with pandemic influenza.

**Results:**

Results of the model over a range of reproduction numbers and effective vaccination coverage confirm this apparent increased risk in the Northern, but not the Southern, hemisphere. This is due to unvaccinated individuals being more likely to be infected with seasonal influenza (if it is circulating) and developing hypothesised temporary immunity to the pandemic strain. Because vaccinated individuals are less likely to have been infected with seasonal influenza, they are less likely to have developed the hypothesised temporary immunity and are therefore more likely to be infected with pandemic influenza. If the reproduction number for pandemic influenza is increased, as it is for children, an increase in the apparent risk of seasonal vaccination is observed. The maximum apparent risk effect is found when seasonal vaccination coverage is in the range 20-40%.

**Conclusions:**

Only when pandemic influenza is recently preceded by seasonal influenza circulation is there a modelled increased risk of pandemic influenza infection associated with prior receipt of seasonal vaccine.

## Background

Recent Canadian research has suggested that individuals who had received the seasonal influenza vaccination were at a higher risk of being infected with pandemic influenza H1N1 2009 (pH1N1) than unvaccinated individuals [1]. Four different studies from Canada reported that, compared to no vaccination, prior vaccination with seasonal vaccine increased the odds of infection with pH1N1 from 1.4 to 2.5. The authors proposed several explanations for these unexpected findings which were further discussed by Viboud and Simonsen [2]. Similar, but weaker, findings were found in several studies from the United States (US) with non-significant odds ratios above 1 [3-5]. In contrast to the Northern hemisphere experience, a study in the Southern hemisphere (Victoria, Australia) found no risk associated with receipt of seasonal vaccine with an age-adjusted odds ratio of 0.97 [6]. All of these studies only used data from the ‘first wave’ of the pH1N1 outbreak covering the period March-July 2009 and hence our model focuses on this time frame as well.

People infected with one strain of influenza will, in general, have immunity to this strain and will have partial immunity (cross-immunity) to strains that emerge by mutation from the infecting strain. The level of cross-immunity will diminish with increasing number of amino acid differences between strains [7]. New pandemic strains are characterized by minimal host immunity. It has been postulated that a ‘short-lived strain transcending immunity’ after any influenza infection may exist [8]. Modelling studies have demonstrated that only with the inclusion of this short-lived strain transcending immunity do the models reproduce the slender phylogenetic tree structure [9] of influenza [8,10-14]. The mean duration of this hypothesised immunity is unclear but values in the range 3-6 months give realistic results in the models, and are supported by the available experimental and epidemiological literature, well summarized in Ferguson et al. 2003 [8].

Although previously demonstrated in animal models (see references in [15]) the concept of heterotypic and heterosubtypic temporary immunity is very difficult to demonstrate in observational studies of humans, because it is rare to find sequential or contemporaneous circulation of different influenza types or sub-types. The 2009 pH1N1 outbreak was one such opportunity. Prior to the pandemic of 2009, the most recent period when this occurred was in 1977 when influenza A(H1N1) followed circulation of influenza A(H3N2). A largely forgotten and inappropriately uncited study from Japan was able to capitalise on the routine collection of sera from Japanese school children between December 1977 and March 1978 to explore temporary heterosubtypic immunity [15]. The investigators used questionnaire data and sequential serological samples from children at four different schools to demonstrate ‘cross-subtype protection in humans during sequential and/or concurrent epidemics caused by two viral sub-types.’ In particular they demonstrated a significantly lower proportion of infections with both sub-types than would have been expected if sub-type infections occurred independently (*p* < 0.005). The authors also showed that of the 16 pupils who were ill twice (that is, reported clinical symptoms and had serological evidence of infection), the probability of dual infection increased with the time since the first infection. Only 4 of the 16 infections occurred in the first two weeks after the primary infection, with the earliest infection occurring 6 days after the first infection. This observation tentatively supports the hypothesis of almost complete temporary immunity at the time of infection with immunity waning over time.

We have previously postulated that the apparent difference in risk from receipt of the seasonal vaccination is due to the timing of the emergence of the pandemic strain relative to the usual seasonal influenza season in the Northern and Southern hemispheres [16]. In particular, if the pandemic occurred soon after the usual influenza season, as it did in the first wave in the Northern hemisphere, recent prior infection with seasonal influenza was protective against pH1N1 infection due to the hypothesis of global temporary immunity, which implies that infection with any strain of influenza confers temporary immunity of 3-6 months duration to infection with any other strain of influenza.

Individuals who had received seasonal vaccine had a lower probability of being infected with the seasonal strain and an apparent greater risk of pH1N1 infection, having forfeited the protection from temporary immunity that seasonal infection would have conferred. In Southern hemisphere Victoria the pandemic strain displaced the seasonal strain and comprised over 99% of all circulating viruses for which sub-typing was available [6,17]. Hence there was no apparent increased risk from receipt of the seasonal vaccine in the Southern hemisphere as there was virtually no seasonal influenza circulating to give temporary immunity to the unvaccinated individuals.

Here we develop a simple mathematical model to demonstrate that the hypothesised temporary strain transcending immunity, together with the timing of the pandemic, give a plausible explanation for the differences seen between the Northern and Southern hemisphere studies on the risk of pH1N1 infection associated with prior seasonal vaccination during the first wave of the outbreak. We have developed the model as a proof of the concept that temporary immunity can possibly explain the conflicting associations with seasonal vaccination, without the need to assume that the vaccine itself is harmful. The model captures the interacting competition of seasonal influenza with a newly introduced pandemic influenza strain but is not intended to be a detailed strain-mutation model that accurately reflects the long term evolution of influenza as recently developed by other researchers [8,11-14,18,19]. The model is deterministic and as such does not incorporate the stochastic effects seen in real epidemics. Given we are interested in simulating situations where the pandemic strain takes off, so that comparison can be made to the actual occurrence, we did not consider stochastic models were necessary. The aim was to keep the model as simple as possible to determine if the strain transcending immunity concept is a possible explanation of the results seen in the vaccination risk studies.

The studies on the risk of the seasonal influenza vaccination were conducted over the first wave of the outbreak (March-July 2009) [1,3-6]. Hence we mostly restrict our model analysis to the case where the pH1N1 strain was introduced soon after the usual influenza season (Northern hemisphere), or slightly preceding or concurrent with the usual influenza season (Southern hemisphere). We postulate that in the Northern hemisphere, in countries that had a sizable first wave of pH1N1 infection in March-July 2009, this apparent risk from the seasonal vaccination will be observed. In contrast, people infected with pH1N1 in the ‘second wave’ (October 2009 onwards) are not expected to show this apparent risk due to the long delay from the previous seasonal influenza season. We are currently not aware of any studies from the second wave of pH1N1 infections to confirm or refute this postulation.

## Method

The model is based on a standard SIR (Susceptible, Infective, Recovered) compartmental model [20]. We allow for two different strains, seasonal (denoted by 1) and pandemic (denoted by 2), vaccination coverage for the seasonal strain, relatively long-lived immunity to the infecting strain and temporary immunity to any strain after infection. Because we consider only a short time span, the population size is considered constant and we can refer to the proportion of people in each class rather than the number in the class in the standard way. In the interests of model simplicity, the population is assumed to be homogeneous and well mixed and we have made no attempt to distinguish between different age classes or different transmission properties in sub-populations. We have assumed that seasonal vaccination provides protection against seasonal influenza infection to a proportion of vaccinated people, depending on vaccine effectiveness and vaccine coverage, but that it provides no protection against infection with pH1N1.

As vaccination alters the susceptibility to the seasonal strains we partition the population into 3 broad groups: the proportion of hosts vaccinated with the seasonal influenza vaccine (susceptible to the pandemic strain only); unvaccinated hosts (susceptible to both pandemic and seasonal influenza); and hosts susceptible to seasonal influenza only. Initially this latter group only comprises individuals who have some cross-immunity to the pandemic strain such as was seen with older people during the pH1N1 2009 outbreak [21]. Because vaccination is not completely effective, we model effective vaccination coverage which we define as vaccination coverage multiplied by the vaccine effectiveness for the seasonal strain. Throughout we let sub/super script 1 refer to seasonal and 2 to pandemic strains. We further divide each of the broad groups into the following classes: susceptible (*S*), infective (*I*), temporarily immune to all strains (*T*) and recovered (*R*). Superscripts are used to identify strain susceptibility type and subscripts the infecting strain. Hence the classes considered are given by

*S*^12^**Unvaccinated**, susceptible to 1 (seasonal) and 2 (pandemic)

*S*^1^ Susceptible to 1 (seasonal) only

*S*^2^**Vaccinated**, susceptible to 2 (pandemic) only

 Was susceptible to 1 and 2 and now infected with 1

 Was susceptible to 1 and 2 and now infected with 2

 Was susceptible to 1 only and now infected with 1

 Was susceptible to 2 only and now infected with 2

 Temporary immune to all strains, was susceptible to 1 and 2 and recently infected with 1

 Temporary immune to all strains, was susceptible to 1 and 2 and recently infected with 2

 Temporary immune to all strains, was susceptible to 1 only and recently infected with 1

 Temporary immune to all strains, was susceptible to 2 only and recently infected with 2

*R* Recovered and immune to 1 and 2

**Figure 1:**
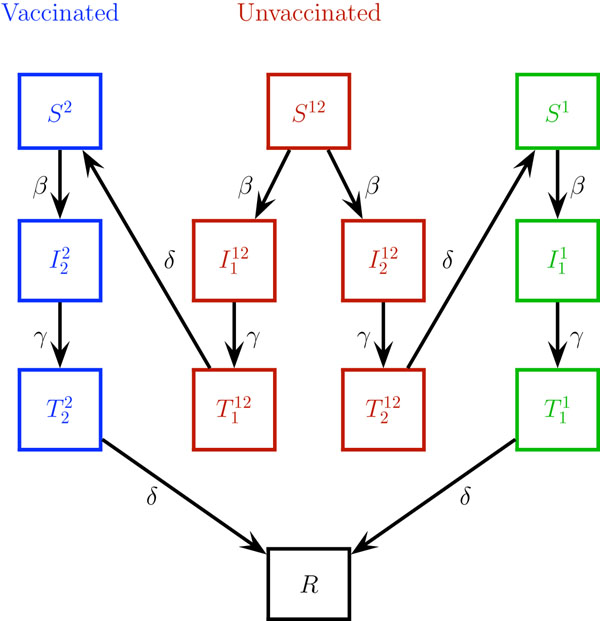
**Possible disease paths through the different classes.** S - susceptible, I - infective, T - temporarily immune to all strains, R - recovered. Sub/superscripts 1 refer to seasonal and 2 to pandemic. Superscripts are strains the individuals was susceptible to, subscripts the strain they are or have recently been infected with. Labels on the links are the relevant rates of progression between classes.

For example, depending on the effective vaccine coverage, a proportion of hosts vaccinated against seasonal influenza will be susceptible only to the new pandemic strain, and will be a member of the *S*^2^ class. Once this host is infected with pandemic influenza he/she will move to the  class and will then recover to be in the temporary immunity  class. Over time the host loses temporary immunity to all strains and moves to the *R* class. Of particular importance in this model are those hosts who are unvaccinated and initially a member of the *S*^12^ class, being susceptible to both seasonal and pandemic influenza. A member of this class who becomes infected with seasonal influenza will then move to the  class, and recover to the  class before moving to the *S*^2^ class. Membership of the  class is important since these hosts have developed temporary immunity to pH1N1 infection. All possible disease progression paths are shown schematically in Figure 1.

For a fixed population size *S, I, T* and *R* can also represent the proportion of the population in each group. If *β* is the transmission rate, *γ* is the disease recovery rate (hence 1/*γ* is the average infectious period) and *δ* the recovery rate from the temporary immunity (hence 1/*δ* is the average length of the immunity period) then the disease dynamics can be modelled by(1)(2)(3)(4)(5)(6)(7)(8)(9)(10)(11)

For example, equation (1) states that unvaccinated individuals (*S*^12^) can be either infected by seasonal influenza infectious individuals  or pandemic strain infectious individuals  equation (3) states that the proportion of individuals only susceptible to the pandemic strain (*S*^2^), which is originally a proportion of vaccinated hosts, can decrease by being infected by pandemic strain infectious individuals and can also increase due to individuals who were originally susceptible to both strains leaving the temporary immunity class after being infected with the seasonal strain  (and hence are susceptible to only the pandemic strain). As the population size is fixed there is no need to keep track of the *R* class.

The system of ordinary differential equations (1)-(11) is solved numerically using MATLAB. Different initial conditions are used depending on the effective vaccination coverage and any prior immunity. Residual cross-immunity from prior infection with related strains is incorporated in the initial conditions. The Canadian studies reported a vaccine coverage of approximately 30% and a vaccine effectiveness of 56% resulting in an effective vaccination coverage of 17%. Other studies report vaccine effectiveness for healthy young adults, the age group most often infected with pH1N1, of approximately 70% [22]. The different timings of the seasonal and pandemic strains are simulated by seeding the population with a small proportion (0.01%) of one strain and at some later time introducing the other strain. Values of *β* and *γ* are chosen to give a basic reproduction number (*R*_0_) [23] typical of influenza of between 1.3 and 1.8. For simplicity it is assumed that both the seasonal and pandemic strains have the same basic reproduction number, consistent with estimates of R for both seasonal [24] and pandemic influenza [25]. The average duration of strain transcending temporary immunity is modelled to be 120 days. The results presented here are relatively insensitive to this parameter, except for the case where there is a long interval between the introductions of seasonal and pandemic influenza.

To compare our results with the observational studies [1,3-6] we calculate the ratio of the odds of pH1N1 infection for vaccinated versus unvaccinated individuals. If *p_v_* is the probability of a vaccinated individual being infected with pH1N1 and *p_u_* is the probability of an unvaccinated individual being infected with pH1N1 then the odds ratio (OR) is defined to be(12)

## Results and discussion

We compare the Southern hemisphere, when pandemic influenza was not preceded by the circulation of seasonal influenza, to the Northern hemisphere where pandemic influenza circulated soon after seasonal influenza.

### Pandemic strain replacing the seasonal strain - Southern hemisphere

In the Southern hemisphere pH1N1 2009 began circulating just prior to the usual winter influenza season which normally spans June to October [26]. In Victoria, Australia the first confirmed case of pH1N1 infection was recorded on 20 May 2009 but there is evidence that the virus may have been circulating in Victoria for up to a month prior to that [27]. Within a very short time pH1N1 was the dominant strain circulating with over 99% of all circulating viruses for which sub-typing was available being pH1N1 [6,17]. A study using sentinel general practice surveillance data indicated that, in the absence of a circulating seasonal strain, seasonal influenza vaccine provided no effective protection against pH1N1 infection [6].

Running the model described above with the pandemic strain introduction 30 days before the seasonal strain, as was probably the case in Victoria [27], and an effective vaccination coverage of just 10%, results in virtually complete domination by the pandemic strain with over 99.0% of cases being the pandemic strain. The effective vaccination coverage in Victoria would have been likely to be of the order of 11%. We estimate this using the vaccine coverage of 22% in the controls of the case control study of vaccine effectiveness [6] and an assumed vaccine effectiveness of 50% [28]. Higher effective vaccination coverage results in even higher dominance of the pandemic strain. If the strains are introduced concurrently, which was more typical in other Australian jurisdictions, the pandemic strain is modelled to comprise over 95% of cases. In these instances, even with the same reproduction numbers for each strain, seasonal vaccination gave the pandemic strain a competitive advantage. As there was very little seasonal influenza circulating the seasonal vaccine had little effect on the odds ratio calculation of the risk of receipt of the seasonal vaccine and so the odds ratio was around 1.

### Pandemic strain circulating after seasonal strain - Northern hemisphere

**Figure 2:**
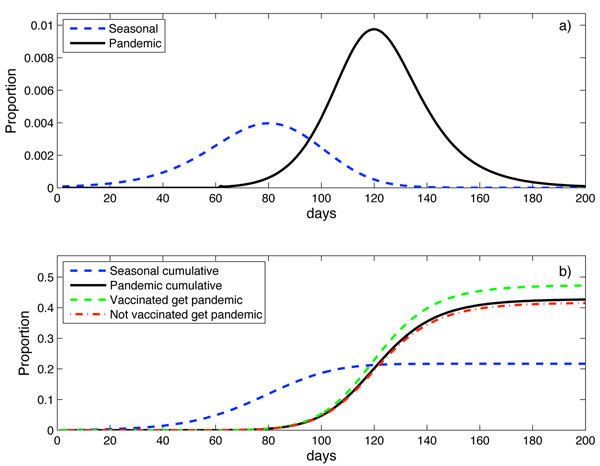
**Pandemic delayed 60 days with a 20% effective seasonal vaccination coverage.** Pandemic strain introduced 60 days after the seasonal strain with a 20% effective seasonal vaccination coverage, a mean temporary immunity of 120 days and *R*_0_ = 1.5. a) Proportion of new infectives each day infected with the seasonal strain (dashed blue line) and the pandemic strain (solid black line). b) Cumulative proportions infected with the seasonal strain (dashed blue line), total infected with the pandemic strain (solid black line), vaccinated individuals infected with the pandemic strain (dashed green line) and proportion of unvaccinated individuals infected with the pandemic strain (dot-dash red line).

In some temperate regions of the Northern hemisphere the pandemic strain began circulating in a first wave during April 2009, near the end of the usual winter influenza season. Figure 2 shows a typical result from the model of this scenario with the pandemic strain introduced 60 days after the seasonal strain, an effective vaccination coverage of 20% and *R*_0_ = 1.5. Figure 2a shows the proportion of newly infected individuals each day. The seasonal strain is shown as the dashed blue line and the pandemic strain as the solid black line. Figure 2b shows the time-dependent cumulative number of cases of each strain with the corresponding lines for seasonal and pandemic influenza as in Figure 2a. Also shown is the proportion of vaccinated individuals (dashed green line) and the proportion of unvaccinated individuals (red dot-dash line) who become infected with pH1N1. The effect of seasonal vaccination on individuals infected with the pandemic strain is evident as the proportion of infected vaccinated individuals is larger than the proportion of infected unvaccinated individuals. The odds ratio of vaccinated versus unvaccinated individuals who were infected with pH1N1 can be calculated for this example as *OR* = 1.35. This apparent increased risk of pH1N1 infection is due to unvaccinated individuals being more likely to have had seasonal influenza infection and have developed temporary immunity to the pandemic strain. This temporary immunity was not seen in a proportion of vaccinated individuals. We do not need to suggest a harmful effect of the vaccine to find the apparent harmful association of receipt of seasonal vaccine and pH1N1 infection.

As described earlier, to concur with the time frame of the vaccine risk studies, we mainly consider the first wave of pH1N1 infection and so mostly restrict the analysis to cases where the delay from the usual influenza season to the pandemic strain introduction is less than 5 months. One case is run for a delay of 240 days to demonstrate the case in the Northern hemisphere in jurisdictions where there was no substantial first wave and only a second wave (for example many parts of Europe).

**Figure 3:**
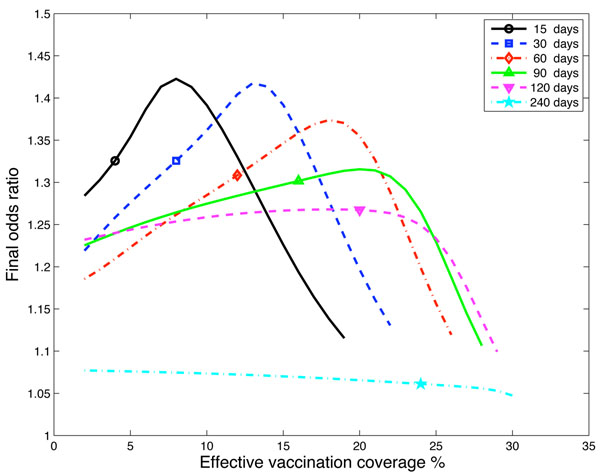
**Odds ratio for six different seasonal strain delays with *R*_0_ = 1.5.** Odds ratio versus effective vaccination coverage for six different delays from seasonal strain introduction to pandemic strain introduction. All calculations are with *R*_0_ = 1.5 and 1/*δ* = 120 days. Delays of 120 days or less correspond to a first wave scenario, the delay of 240 days corresponds to a second wave only scenario. The symbols on the lines are to aid in differentiating the line types and not a specific data point of interest.

Of interest is the effect on the odds ratio of the timing of the introduction of the pandemic strain and the level of effective vaccination coverage. In particular, the model supports the results found in the studies from the USA of a weak, but not statistically significant risk, [3-5] and the results from Canada [1] of significant risks, illustrated by odds ratio estimates ranging from 1.4 to 2.5. Figure 3 is a plot of the odds ratio versus the effective vaccination coverage for 6 different delays (15, 30, 60, 90, 120 and 240 days) from the introduction of the seasonal strain to the introduction of the pandemic strain. Values are plotted when both the seasonal and pandemic strains had a cumulative incidence of at least 5% of the susceptible population. This ensures that both epidemics are large enough to make calculation of the odds ratio reliable. The largest odds ratio calculated was 1.42 for the shorter delays. Longer delays are associated with lower modelled odds ratios, a reflection of waning temporary immunity although there is still a consistent odds ratio around 1.25 even with a delay of 120 days. Delays of up to 120 days correspond to a first wave scenario. Also included in Figure 3 are the results for a long delay of 240 days consistent with jurisdictions that had no significant pH1N1 first wave but a significant second wave. In this case the odds ratio was around 1.07 which is substantially lower than for the shorter delays. This suggests that in those jurisdictions the apparent risk from the seasonal vaccination may not be observed since the odds ratio is close to 1. We are not aware of any studies published that investigate this second wave scenario.

As demonstrated in Figure 3 for each delay less than or equal to 120 days there is an effective vaccination coverage for which the odds ratio is a maximum. This is the effective vaccination coverage where the apparent risk of the seasonal vaccination is strongest. As the effective vaccination coverage increases beyond this maximum point the seasonal epidemic is smaller due to the higher vaccination coverage. This smaller epidemic size means the proportion of unvaccinated individuals infected with the seasonal strain also decreases. The result of this is that the odds ratio decreases as the effective vaccination coverage increases in this region. When the effective vaccination coverage is increased further, the seasonal epidemic does not take off and hence vaccinated and unvaccinated individuals appear almost the same and the odds ratio tends to one. For all delays there is no modelled increased risk from vaccination when the effective vaccination coverage is above 30%, since effective vaccine coverage at this level (for example, 50% coverage with a vaccine that was 60% effective or 60% coverage with a vaccine that was 50% effective) aborts the seasonal epidemic.

**Figure 4:**
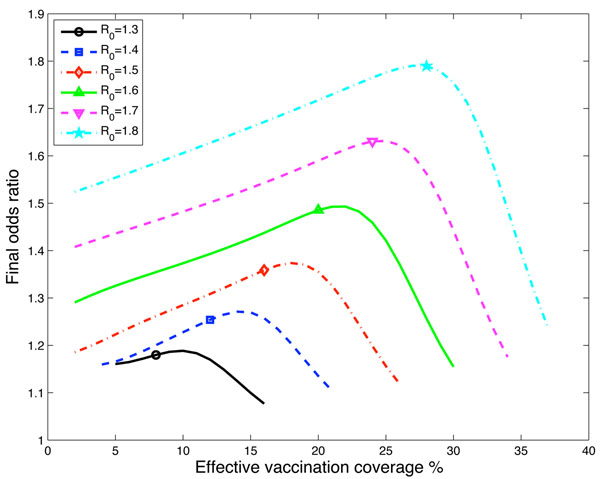
**Odds ratio for six different *R*_0_ values with 60 day delay**. Odds ratio versus effective vaccination coverage for six different *R*_0_ values. All calculations are with 1/*δ* = 120 days and the delay from seasonal strain introduction to pandemic strain introduction of 60 days. The symbols on the lines are to aid in differentiating the line types and not a specific data point of interest.

The odds ratio of vaccinated versus unvaccinated individuals also depends on the disease reproduction number. Shown in Figure 4 is the odds ratio versus effective vaccination coverage for 6 different basic reproduction numbers ranging from 1.3 to 1.8 [25] for a pandemic introduction delay of 60 days. The higher the reproduction number the higher the odds ratio and hence the greater apparent risk from the seasonal vaccination. This pattern is also seen over all delays considered from 15 to 240 days.

In the Canadian studies [1] vaccination coverage of around 30% was reported and vaccine effectiveness was estimated as 56% giving an effective vaccination coverage of approximately 17%. This is the region where there is maximum effect of the seasonal vaccination for a delay of the pandemic after the seasonal circulation of 60 to 90 days. In Canada the peak incidence of seasonal influenza occurred 11 weeks (77 days) before the first notified cases of pH1N1. The model gives maximum value of the odds ratio from 1.15 to 1.75 over the range of plausible reproduction numbers, delay between the introduction of seasonal and pandemic influenza and effective vaccination coverage. These estimates are consistent with the lower end of estimates from the Canadian studies. However, if the cases of pandemic influenza were predominantly in children, the reproduction number could be higher than 1.8 [29]. In the Canadian studies, the Quebec sample comprised 44% children with pH1N1 infection and the Ontario study 61%. If we used a value of *R* = 2.0 in our model, consistent with values reported for school children in Japan [29], with a delay of 70 days and effective vaccination coverage of 17%, both consistent with observations from Canada, we estimate an odds ratio of 2.0 for the risk of pH1N1 infection following receipt of seasonal vaccine.

Over a wide range of parameter values the maximum odds ratio occurs for effective vaccination coverage in the range 15-25%. This range will be generated if vaccination coverage is 20-40% and vaccine effectiveness is 50-70% [22,28]. We could therefore expect to see an apparent harmful effect of vaccination in the Northern hemisphere first wave, even though we assumed that the vaccine provided neither harm nor protection, when seasonal vaccine coverage was in the range 20-40% and seasonal influenza preceded pandemic influenza circulation.

When the pandemic occurs after circulation of the seasonal strain, the maximum odds ratio does not occur until near the end of the pandemic since the cumulative number of cases with pH1N1 infection affects the odds ratio in an ongoing manner. To obtain an accurate measure of the association between seasonal vaccination and pH1N1 infection, it is necessary to measure the final odds ratio once the pandemic is over. The US studies that showed only a weak risk associated with vaccination [3-5] used data only up to early May or June, which was before the end of the first wave of the pandemic. These odds ratios may be low because of this but could still be consistent with our hypothesis.

## Conclusions

Our simple strain competition model incorporating a temporary strain transcending immunity gives results consistent with findings of the association between receipt of seasonal influenza vaccine and the risk of pH1N1 infection in both the Northern and Southern hemispheres. Our model was predicated on the assumption that seasonal vaccine had no effect on the risk of pH1N1 infection. The apparently conflicting results, where some studies from the Northern hemisphere reported an apparently harmful association of receipt of seasonal vaccination with pH1N1 infection, while neither benefit nor harm was found in the Southern hemisphere, can possibly be explained by temporary immunity and the timing of the circulation of seasonal and pandemic influenza infection, without the need to suggest a harmful effect of the vaccine itself. Although it should be noted that the concept of temporary immunity is conjecture at this stage, it is supported by the study of Japanese school children when influenza A(H1N1) circulated soon after A(H3N2) [15]. Our model showed that seasonal vaccination appeared to increase the risk of pH1N1 infection only in the Northern hemisphere, when seasonal influenza preceded pandemic influenza circulation, and only due to a proportion of the population having recently been infected with seasonal influenza developing temporary immunity to pH1N1 infection. This only applies to the first wave of pH1N1 in the Northern hemisphere and is unlikely to apply to the second wave when the delay from seasonal influenza season to the introduction of the pandemic strain is considerably longer.

The maximum effect was modelled to occur with seasonal vaccination coverage in the range of 20-40%. The apparent risk increased with increasing reproduction number. A section of the population with a higher intrinsic reproduction number, such as children, would therefore appear to have had a higher risk. Our model, incorporating the hypothesized concept of temporary immunity, reproduced risk estimates very similar to those reported without needing to suggest that the vaccine itself was harmful.

If the strain-transcending temporary immunity concept is correct then other Northern hemisphere jurisdictions that had a sizable first wave of pH1N1 infection (for example the United Kingdom) would be expected to show an increased apparent risk of the seasonal vaccination. We know of no published studies from the United Kingdom to confirm or refute this at present. Further vaccination risk studies covering a wider time interval incorporating the Northern hemisphere second wave are eagerly awaited and will provide further information. For example, if a Canadian study of second wave pH1N1 infections found no apparent risk due to seasonal vaccination then that gives some additional weight to the temporary immunity hypothesis, alternatively if a similar apparent risk is found then an alternative mechanism is more likely to be involved.

## Competing interests

The authors declare that they have no competing interests.

## Authors' contributions

GM refined the hypothesis, developed the model, wrote computer code, wrote and edited the manuscript. SB refined the hypothesis, helped to develop the model, wrote and edited the manuscript. HK conceived the study, suggested the hypothesis, provided details on influenza epidemiology, wrote and edited the manuscript.
